# Promoting Alcohol Reduction in Non-Treatment Seeking parents (PAReNTS): a pilot feasibility cluster randomized controlled trial of brief alcohol interventions with parents in contact with child safeguarding services

**DOI:** 10.1093/alcalc/agad076

**Published:** 2023-11-09

**Authors:** Ruth McGovern, Deborah Smart, Hayley Alderson, Tony Fouweather, Eileen Kaner

**Affiliations:** Population Health Sciences Institute, Newcastle University, Newcastle upon Tyne NE2 4AX, United Kingdom; Population Health Sciences Institute, Newcastle University, Newcastle upon Tyne NE2 4AX, United Kingdom; Population Health Sciences Institute, Newcastle University, Newcastle upon Tyne NE2 4AX, United Kingdom; Population Health Sciences Institute, Newcastle University, Newcastle upon Tyne NE2 4AX, United Kingdom; Population Health Sciences Institute, Newcastle University, Newcastle upon Tyne NE2 4AX, United Kingdom

**Keywords:** alcohol, brief intervention, parents, social care, pilot feasibility trial

## Abstract

Many parents who come into contact with early help and children’s social care services are risky drinkers. This study aimed to investigate the feasibility and acceptability of conducting a trial of brief alcohol interventions within this setting. We conducted a three-arm pilot feasibility cluster randomised controlled trial in the North-East of England. The additive interventions were: i) screening and a healthy lifestyle leaflet (control); ii) brief advice; iii) extended brief intervention. The trial was later reduced to two-arm due to the extended brief intervention being infeasible. Of the 1769 parents that were approached, 429 consented to be screened (24%), the majority were eligible to participate (*n* = 415; 97%), 147 of which (35%) scored ≥5 on the AUDIT-C screening tool. There were 108 parents (74%) who consented to participate in the trial (*n* = 50 control; *n* = 58 brief advice). Follow-up rates at 6 and 12-months were 61% and 43%. The TLFB30 was found to be a suitable tool to measure the primary outcome of heavy episodic drinking. Qualitative data showed that parents and practitioners largely found trial procedures to be acceptable, however, care should be taken when discussing alcohol risk with parents in this setting. Most of the a-priori success criteria were met in this pilot feasibility trial. The findings suggest that it may be feasible to conduct a two-arm randomised controlled trial of brief alcohol interventions to parents in contact with early help and social care. The TLFB30 was found to be a suitable tool to measure the primary outcome of heavy episodic drinking.

## Teaser

**Short Summary:** It may be feasible to conduct a pilot feasibility study of brief alcohol advice compared to control with risky drinking parents in contact with early help and children’s social care services. Care should be taken when discussing the risks (particularly to children) with parents in this setting.

## Introduction

Parental risky alcohol use is a substantial public health (World Health Organisation 2014) and child protection (Canfield *et al*. 2017) concern worldwide. Research estimates that between 5% and 30% of children in European countries (EMCDDA 2010) and 10.5% of children aged under 17 years in USA (7.5 million) live with at least one “risky drinking” parent (Lipari and Van Horn 2017). Risky drinking can be defined as a pattern of consumption that increases the risk of social, legal, health, occupational, and economic problems for the user (Babor *et al*. 2001). Having a parent who is a risky alcohol user is also often associated with a range of negative outcomes (McGovern *et al*. 2020) and experiences (Muir *et al*. 2022) in children. As a result, risky parental alcohol use is identified as an area of need in 14% of all child in need assessments in England (Department for Education 2022) and may co-occur and cause accumulative stress for families (Muir *et al*. 2022). Successive major reviews of UK child protection services highlighted the importance of preventive rather than reactive services as being more effective in improving child welfare (Munro 2011; MacAlister 2022). Despite this, there is no established preventative intervention for parental risky alcohol use (McGovern *et al*. 2021; McGovern *et al*. 2022a).

A large amount of high-quality evidence has accumulated in support of brief alcohol interventions, although this evidence generally relates to primary care (Kaner *et al*. 2016) and other health care settings (Wilson *et al*. 2012; Schmidt *et al*. 2016). This evidence-base informed National Institute for Health and Care Excellence recommendations that brief alcohol interventions should be implemented in a range of settings in the UK including social care (National Institute for Health and Clinical Excellence 2010). However, there is a paucity of studies examining the effectiveness of alcohol screening and brief interventions within social care settings or specifically its use with parents where they are in contact with child safeguarding services (Schmidt *et al*. 2014). Early help and children’s social care practitioners are likely to regularly support families affected by parental risky alcohol use within their routine practice, and therefore the delivery of brief alcohol intervention within these settings may provide an opportunity to respond to the alcohol-related needs of this population (Schmidt *et al*. 2014). However, little is understood about the feasibility of delivering brief alcohol interventions within child safeguarding services and whether the approach is acceptable to both practitioner and parents. Practitioners report experiencing problems identifying parental risky alcohol use (Galvani *et al*. 2013). They are often hesitant in initiating conversations about alcohol with parents (Dance *et al*. 2014) and highlight that within a context of safeguarding children, discussing alcohol may jeopardize an already difficult relationship (Hafford-Letchfield *et al*. 2017). Further, parents themselves may be resistant to conversations about alcohol (Forrester *et al*. 2012), may under-report risk (Meinck *et al*. 2016) or may refuse to engage with alcohol interventions that they do not think are relevant to them (Forrester and Harwin 2006). The Promoting Alcohol Reduction in Non-Treatment Seeking parents (PAReNTS) pilot feasibility trial aims to investigate whether it is possible to recruit parents involved with early help family support (family agree to support voluntarily) and/or children’s social care (statutory intervention) who are risky alcohol users, and whether these parents can be retained at 12-month follow-up. The feasibility and acceptability of the brief interventions and trial procedures will also be examined, to inform the protocol of a definitive trial.

## Methods

The PAReNTS trial is a pilot feasibility cluster randomized controlled trial in the North-East of England (ISRCTN60291091). A published trial protocol outlines all of these procedures in detail (McGovern *et al*. 2018). A favorable ethical opinion was granted by the Health Research Authority Social Care Research Ethics Committee (16/IEC08/0037) on 10 November 2019.

### Recruitment

There were three distinct recruitment phases within the trial. These were:

Phase 1: Adhered fully to the published protocol. Participating practitioners within early help and children’s social care services were asked to screen all eligible parents on their caseload using the AUDIT-C screening tool within their routine appointments. Practitioners invited parents who score ≥5 (Bush *et al*. 1998) to participate in the trial. Due to low screening activity by practitioners, it was apparent that the recruitment target for the trial would not be met (further details provided in findings section and Table 4). As such, recruitment to the trial was suspended and a substantial amendment to the recruitment procedures was submitted to the Health Research Authority Social Care Research Ethics Committee.

Phase 2: Parents were approached by the participating practitioners within their routine appointments. The practitioner provided a participant information leaflet to the parent and asked for consent to share their contact details with the research team, to discuss the research further. Parents who gave consent were contacted by the research team who then followed the recruitment procedure described above. Due to low numbers of practitioners approaching parents, it was apparent that the recruitment target for the trial would not be met. This was further compounded by the COVID-19 national lockdown. As such, recruitment to the trial was suspended and a further substantial amendment was submitted to the Health Research Authority Social Care Research Ethics Committee.

Phase 3: Parents received a postal invitation from the local authority to participate in the trial. Invitation letters were sent direct to parents along with a participation information leaflet, AUDIT-C questionnaire, name and contact details slip and a pre-paid envelope. Parents who wished to participate were asked to return the completed AUDIT-C and contact details slip. All responding parents received £10 for completing the AUDIT-C. Researchers then contacted parents who scored ≥5 on the AUDIT-C and invited eligible parents to participate in the trial.

### Inclusion and exclusion criteria

To be eligible, parents must have been 18 years or over and able to give informed consent in English. A broad definition of “parent” was applied wherein any adult who fulfills a parenting role (including step parent, foster parent, or other relative/caregiver) for a child aged 0–17 years was considered eligible for the study. Parents who were attending drug and alcohol services, who had severe, chronic, or acute mental health problems or were severely distressed or whose child is placed on an emergency protection order were ineligible to participate in the study.

### Interventions

One of three interventions were randomly assigned in 1:1 ratio using block randomization. The three (additive) trial arms were:

- Control intervention: Screening and a healthy lifestyle leaflet.
- Brief advice: 20 min of semi-structured alcohol advice adapted for parents.
- Extended brief intervention: Invitation to attend an appointment with a local alcohol treatment service for 40 min of brief lifestyle counseling adapted for parents followed by an optional booster session.

Within Phase 1, none of the randomized parents opted to attend the extended brief intervention appointment and it was no longer possible to achieve the success criteria for this intervention. A substantial amendment was submitted to the Health Research Authority Social Care Research Ethics Committee. The amendment resulted in the trial being reduced to a two-arm trial (control versus brief advice) to avoid unnecessary participant burden.

### Primary outcome measures

Key outcome measures for the pilot feasibility trial were:

(i) Percentage of eligible participants consenting and enrolled at baseline.
(ii) Percentage of enrolled participants followed-up at 12-months.

**Figure 1:**
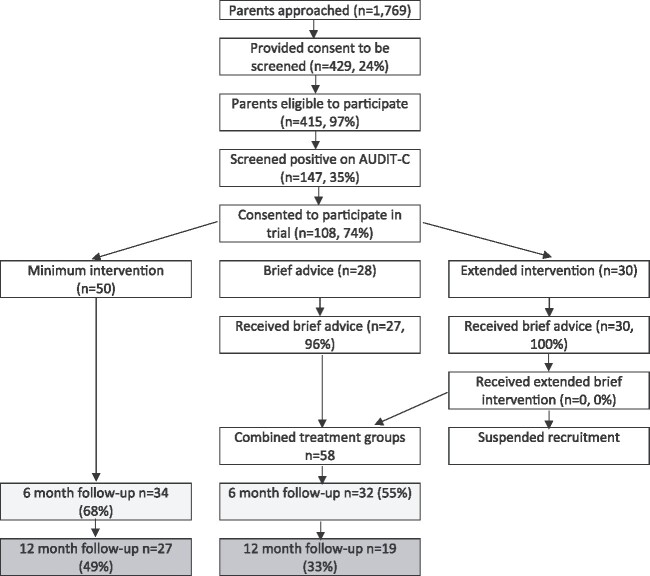
Trial consort diagram

Fig. 1 reports the trial consort diagram.

### Secondary outcome measure

A number of tools were administered at baseline, 6 and 12-month follow-up to assess response variability:

(i) The 30 day Timeline Follow-Back (TLFB30) was the intended primary outcome for a definitive trial (12-months follow-up only) (Sobell and Sobell 1995)
(ii) *Alcohol Use Identification Test* (AUDIT), 10 item tool: AUDIT scores were categorized as 0–7 (low-risk drinking); 8–15 (hazardous drinking); 16–19 (harmful drinking); 20–40 (probable dependent drinking) (Saunders *et al*. 1993)
(iii) *Alcohol problems questionnaire*—this is a clinical instrument for measuring alcohol-related problems (Drummond 1990)
(iv) EQ-5D-5L measured Health-Related Quality of Life (Janssen *et al*. 2013)
(v) Family functioning was assessed with the *Family Perceptions Scale* (FPS), The FPS is a 29-item tool across five domains (nurture, problem solving, expressed emotion, behavioral boundaries and responsibility) (Tiffin *et al*. 2011)
(vi) Mental health and well-being were assessed using the *Warwick Edinburgh Mental Well-Being Scale* (WEMWBS): 14-item scale of mental well-being covering subjective well-being and psychological functioning. A score of 41–44 is indicative of possible mild depression; <41 is indicative of probable clinical depression. The tool has been used extensively with adults (Clarke *et al*. 2011)
(vii) A modified *Client Service Receipt Inventory* (CRI) to assess service usage for economic analysis (Chisholm *et al*. 2000)

### Success criteria

A formal power calculation was not required for this pilot feasibility trial (McGovern *et al*. 2018). A minimum number of 35 participants per study arm at 12-months was recommended to estimate a parameter for a definitive trial (Lancaster *et al*. 2004). An a-priori success criteria consisted of: recruitment of three Local Authorities as research sites within North-East England; ≥60% of eligible participants consenting to pilot feasibility trial; ≥60% of consenting participants accepting/attending intervention sessions; retention of ≥60% of consented participants for provision of key outcome data at 12-months. Where marginal results were reached, a process for decision making after pilot and feasibility trials was followed (Bugge *et al*. 2013); systematically appraising the problems and potential solutions within the linked process evaluation. We assessed item completion rates for study outcomes, including relevant economic data. Acceptability was determined via an interpretive assessment of qualitative interview work with both participating parents and practitioners.

### Statistical and economic analysis

No formal hypotheses were tested. Descriptive analyses were conducted in response to the primary outcomes described above by a researcher who was blinded to intervention allocation at baseline and (where relevant) also at the 6- and 12-month follow-ups. The statistical and economic evaluation tested the feasibility of proposed methods for a definitive trial and included reporting of the proportion of missing data on questionnaires.

### Qualitative process evaluation

We conducted qualitative interviews with purposive samples of practitioners and parents to examine the acceptability of trial procedures. Both participant groups were sampled by local authority area, randomized group and gender. Parents were additionally sampled by AUDIT-C score and practitioners by the number of cases they recruited to the trial. All interviews were conducted using a semi-structured topic guide and were audio-recorded and transcribed verbatim. Anonymised transcribed narrative accounts were used to enable thematic analysis of key issues for participants.

## Results

Of the 1769 parents that were approached, 429 consented to be screened (24%). This varied however by recruitment approach (100% of parents approached during Phase 1 consented to screening; 62% within Phase 2 and 14% in Phase 3). The majority of these parents were eligible to participate (*n* = 415, 97%) and 147 parents (35%) scored five or above on the AUDIT-C screening tool, indicating increasing risk or higher risk drinking. Subsequently, 108 parents consented to participate in the trial (74%). This again varied by the recruitment approach (98% within Phase 1; 96% in Phase 2; and 53% Phase 3).

### Primary outcomes

Within Phase 1, practitioners recruited parents and provided the randomized intervention. We trained *n* = 64 practitioners in trial procedures. The practitioners recruited a total of 45 participants (*n* = 8 control; *n* = 7 brief advice; *n* = 30 extended brief intervention). Of the 64 practitioners, *n* = 24 recruited 1 or more parents to the trial (mean 1.88; range 1–7 parents). Due to changes in the recruitment process which came into effect in Phase 2, three members of the research team recruited all participants in Phase 2 and 3 and provided the randomized intervention. There was a total of *n* = 21 participants recruited in Phase 2 (*n* = 16 control; *n* = 5 brief advice) and 42 participants were recruited in Phase 3 (*n* = 26 control; *n* = 16 brief advice).

### Baseline characteristics

Table 1 describes the demographic characteristics of the participants. The majority of the sample were female (79%), white (93%), the mean (SD) age was 38.9 (8.9), and had a mean of 3.1 children (range 1–8). While female participants were consistently recruited in higher numbers across the recruitment phases, a higher proportion of males were recruited within Phase 3 (31%), than in Phase 1 (16%) or Phase 2 (14%). Age and ethnicity did not vary by recruitment phase. While all participants scored ≥5 on the AUDIT-C (cut-off for risky drinking), when the remaining items of the full AUDIT was administered, 34% of the parents screened below eight that is indicative of low-risk drinking; 44% were hazardous drinkers; 11% were harmful drinkers and 10% were potentially dependent. The mean (SD) AUDIT score of participants within Phase 3 was higher; 5.5 (12.5) than in Phase 1; 11.2 (10.0) and Phase 2; 10.1 (7.5). A total of 37% of the participants reported scores that indicated possible or probable depression on the WEMWEBS. The mental health status of the participants was consistent across each of the recruitment phases.

**Table 1:** Baseline characteristics.

**Characteristics**	**Total cases *n = 108 n/%***	**Control *n = 50 n/%***	**Intervention *n = 58 n/%***
Age	38.9 m (8.9 sd)	40.2 m (7.3 sd)	37.8 m (9.9 sd)
Male (all)	23 (21%)	6 (12%)	17 (29%)
Female	85 (79%)	44 (88%)	41 (71%)
By recruitment phase	Phase 1	Phase 2	Phase 3
Male	7 (16%)	3 (15%)	13 (31%)
Female	38 (84%)	18 (86%)	29 (69%)
Ethnicity			
White	97 (98%)	43 (98%)	54 (98%)
Status			
Married	23 (26%)	9 (24%)	14 (26%)
Living with partner	15 (15%)	7 (16%)	8 (14%)
Single	48 (47%)	23 (51%)	25 (44%)
Separated	7 (7%)	1 (2%)	6 (11%)
Divorced	4 (4%)	3 (6%)	1 (2%)
Widowed	2 (2%)	0 (0%)	2 (4%)
No of children	3.1 m (1.6 sd)	3.3 m (1.7 sd)	2.9 m (1.5 sd)
No of <18 year children	2.4 m (1.5 sd)	2.6 m (1.7 sd)	2.3 m (1.3 sd)
No of resident children <18 year	2.0 m (1.4 sd)	2.2 m (1.5 sd)	1.9 m (1.3 sd)
Smokers	51 (51%)	20 (45%)	31 (55%)
Education			
Further education	50 (49%)	26 (58%)	24 (42%)
Degree	11 (11%)	6 (13%)	5 (9%)
Employment			
Employed full-time	15 (15%)	6 (13%)	9 (16%)
Employed part-time	14 (14%)	11 (24%)	3 (5%)
Self-employed	4%	3 (6%)	2 (4%)
Homemaker	23%	10 (22%)	13 (23%)
Sick	15%	4 (9%)	12 (21%)
Carer	10%	5 (11%)	6 (11%)
Seeking employment	12%	5 (11%)	7 (13%)
other	6%	0 (0%)	3 (5%)
**AUDIT-C (all participants)**	7.12 m (2.0 sd)	6.8 m (1.9 sd)	7.5 m (2.0 sd)
Phase 1 recruitment *n* = 45	7.1 m (1.8 sd)		
Phase 2 recruitment *n* = 21	7.1 m (2.2 sd)		
Phase 3 recruitment *n* = 42	7.3 m (2.1)		
**AUDIT (10 items) (all participants)**	11.1 m (6.0 sd)	11.3 m (5.9 sd)	11.0 m (6.2 sd)
<8 (low risk)	37 (34%)	21 (42%)	16 (28%)
8–15 (hazardous drinking)	47 (44%)	16 (32%)	31 (53%)
16–19 (harmful drinking)	12 (11%)	6 (12%)	10%
20–40 (probable dependent)	11 (10%)	7 (14%)	4 (7%)
**AUDIT (10 item) (by recruitment phase)**	**Phase 1**	**Phase 2**	**Phase 3**
<8 (low risk)	15 (34%)	6 (29%)	16 (38%)
8–15 (hazardous drinking)	22 (50%)	10 (48%)	15 (38%)
16–19 (harmful drinking)	5 (11%)	2 (10%)	5 (12%)
20–40 (probable dependent)	2 (5%)	3 (14%)	6 (14%)

### Follow-up

Follow-up rates were 61% at 6-months and 43% at 12-months. The approach taken by the research team and the rate of retention varied by recruitment phase. In Phase 1, we primarily followed-up participants by telephone as this has been found to result in lower attrition in other brief alcohol intervention trials with disadvantaged populations (Addison *et al*. 2018). This approach achieved only 21% follow-up at 12-months. In Phase 2, we made three attempts to contact the participants by telephone before reverting to postal questionnaire, achieving 46% follow-up at 12-months. In Phase 3 however we relied entirely on postal questionnaire and achieved 63% follow-up at 12-months.

The planned primary outcome measure within a definitive trial is frequency of heavy episodic alcohol use as measured by the TLFB. The majority (93%) provided a response to the TLFB at 12-months follow-up. The control group reported a higher mean (SD) frequency of heavy episodic alcohol use than the intervention group 4.4 (7.8) against 2.9 (3.4) and alcohol-related problems (APQ) 3.6 (3.2) against 3.0 (3.1) at 12-months follow-up. More participants in the control group reported mental health concerns at 12-months follow-up (WEMWBS) including probable clinical depression (48% against 28%). Both groups reported similar overall health (EQ-5D-5L). Participants in both control and intervention groups evaluated their family functioning (FPS) within normative ranges at all time points (see Table 2).

**Table 2:** Descriptive baseline follow-up data (6 & 12 month).

	**CONTROL**			**INTERVENTION**		
**VARIABLE**	Baseline	6 m	12 m	Baseline	6 m	12 m
**TLFB** (intended primary outcome measure)			4.4 (7.8)			2.9 (3.4)
Missing			2			1
**AUDIT (10 items)**	11.3 (5.9)	5.3 (7.0)	9.0 (7.6)	11.0 (6.2)	5.5 (7.2)	8.1 (4.9)
Missing	0	2		1	0	0
**Weekly spend/alc**	£11.61 (9.81)	£13.22 (10.67)	£16.92 (14.94)	12.79 (10.99)	£14.07 (17.42)	£9.7 (8.88)
Missing	15 (30%)	19 (56%)	15 (56%)	11 (19%)	7 (22%)	9 (47%)
**APQ**		3.7 (4.8)	3.6 (3.2)		4.3 (3.8)	3.0 (3.1)
Missing		0 (0%)	0 (0%)		0 (0%)	0 (0%)
**FPS**						
*Nurture*	20.6 (3.6)	12.8 (9.9)	20.3 (2.8)	20.3 (3.1)	11.1 (10.5)	18.9 (6.5)
*Problem solving*	19.0 (3.8)	14.5 (10.9)	21.9 (3.2)	18.4 (3.1)	12.1 (11.4)	20.4 (6.9)
*Expressed emotion*	11.1 (3.5)	7.9 (6.5)	10.9 (3.4)	12.1 (4.0)	6.5 (6.6)	11.2 (4.8)
*Behavior boundaries*	15.2 (3.2)	9.9 (7.5)	14.6 (3.0)	14.6 (3.2)	8.5 (8.2)	14.0 (5.0)
*Responsibilities*	14.0 (3.7)	8.8 (7.0)	13 (3.2)	13.6 (3.5)	7.6 (7.6)	13.2 (5.5)
*Communication*	28.8 (4.7)	27.0 (4.0)	28.9 (4.1)	27.3 (4.5)	26.7 (4.2)	27.7 (5.2)
Total FPS score	57.6 (13.6)	39.0 (30.0)	58.9 (11.5)	54.9 (12.3)	32.8 (32.4)	55.2 (22.8)
Missing data	5 (10%)	10 (29%)	1 (4%)	0 (0%)	3 (9%)	2 (22%)
**EQ-5D** (*n*, %) *Mobility*						
I have no problems walking about	35 (70%)	20 (63%)	17 (63%)	40 (69%)	18 (58%)	14 (74%)
I have slight problems walking about	8 (16%)	3 (9%)	8 (30%)	10 (17%)	7 (23%)	0 (0%)
I have moderate problems walking about	5 (10%)	6 (19%)	1 (4%)	5 (8%)	4 (13%)	3 (16%)
I have severe problems walking about	2 (4%)	3 (9%)	1 (4%)	2 (3%)	2 (7%)	2 (11%_
I am unable to walk about	0 (0%)	0 (0%)	0 (0%)	1 (2%)	0 (0%)	0 (0%)
*Self-care*						
I have no problems washing/dressing myself	45 (90%)	23 (72%)	22 (82%)	52 (90%)	27 (84%)	14 (74%)
I have slight problems washing/dressing myself	1 (2%)	4 (13%)	2 (7%)	2 (4%)	2 (6%)	1 (5%)
I have moderate problems washing/dressing myself	4 (8%)	5 (16%)	2 (7%)	2 (4%)	1 (6%)	3 (16%)
I have severe problems washing/dressing myself	0 (0%)	0 (0%)	0 (0%)	1 (2%)	1 (3%)	1 (5%)
I am unable to wash or dress myself	0 (0%)	0 (0%)	1 (4%)	1 (2%)	0 (0%)	0 (0%)
*Usual activities*						
I have no problems doing my usual activities	37 (74%)	18 (56%)	16 (59%)	40 (70%)	20 (63%)	13 (68%)
I have slight problems doing my usual activities	9 (18%)	6 (19%)	5 (19%)	12 (21%)	7 (22%)	2 (11%)
I have moderate problems doing my usual activities	3 (6%)	5 (16%)	4 (15%)	4 (7%)	3 (9%)	1 (5%)
I have severe problems doing my usual activities	0 (0%)	2 (6%)	0 (0%)	1 (2%)	2 (6%)	3 (16%)
I am unable to perform my usual activities	1 (2%)	1 (3%)	2 (7%)	0 (0%)	0 (0%)	0 (0%)
*Pain/discomfort*						
I have no pain/discomfort	25 (50%)	16 (50%)	11 (41%)	34 (60%)	12 (39%)	7 (37%)
I have slight pain/discomfort	7 (14%)	5 (16%)	8 (30%)	11 (19%)	6 (19%)	6 (32%)
I have moderate pain/discomfort	11 (22%)	6 (19%)	4 (15%)	7 (12%)	9 (29%)	3 (16%)
I have severe pain/discomfort	7 (14%)	3 (9%)	2 (7%)	4 (7%)	4 (13%)	3 (16%)
I have extreme pain/discomfort	0 (0%)	2 (6%)	2 (7%)	1 (2%)	0 (0%)	0 (0%)
*Anxiety/depression*						
I am not anxious/depressed	16 (32%)	12 (38%)	7 (26%)	27 (47%)	11 (34%)	10 (53%)
I am slightly anxious/depressed	15 (30%)	9 (28%)	7 (26%)	12 (21%)	8 (25%)	4 (21%)
I am moderately anxious/depressed	14 (28%)	4 (13%)	8 (30%)	11 (19%)	7 (22%)	3 (16%)
I am severely anxious/depressed	3 (6%)	6 (19%)	4 (15%)	6 (11%)	4 (13%)	1 (5%)
I am extremely anxious/depressed	2 (4%)	1 (3%)	1 (4%)	1 (2%)	2 (6%)	1 (%%)
Overall health today	67 (22.6)	62.8 (21.7)	66.3 (18.4)	68.9 (19.2)	70.7 (15.8)	66.5 (25.4)
**WEMWEBS**	45.5 (12.6)	46.1 (10.8)	44.3 (9.5)	47.6 (9.6)	45 (13.9)	44.5 (14.1)
*No mental health concerns n, %*	26 (54%)	16 (50%)	11 (41%)	38 (68%)	19 (59%)	10 (56%)
*Possible mild depression n, %*	9 (19%)	7 (22%)	3 (11%)	6 (11%)	3 (9%)	3 (17%)
*Probable clinical depression n, %*	13 (27%)	9 (28%)	13 (48%)	12 (21%)	10. 31%	5 (28%)
Missing	2 (4%)	2 (6%)	0 (0%)	2 (3%)	0 (0%)	1 (5%)

Participants reported low service usage across most service types, across groups, and time. The most common service accessed by parents was GP, early help/children’s social care and outpatient services, while children mostly accessed outpatient care, early help/children’s social care and child and adolescent mental health services. Children and parents reported most frequent contact with early help/children’s social care services. There was a large number of missing data relating to service usage (see Table 3).

**Table 3:** Service usage data.

	**Total number of participants reporting usage**		**Missing data**		**Number contacts—mean**	
**Adult service use**	6 m (*n* = 66)	12 m (*n* = 46)	6 m	12 m	6 m	12 m
Inpatient episodes	1 (2%)	1 (2%)	3 (5%)	2 (4%)	0.0 (0.1)	0.02 (0.2)
Outpatient	8 (12%)	3 (7%)	8 (12%)	4 (9%)	0.1 (0.4)	0.07 (0.3)
Early help/children’s social care	15 (23%)	4 (9%)	10 (15%)	6 (13%)	3.66 (9.6)	0.93 (3.0)
Community mental health services	3 (5%)	1 (2%)	8 (12%)	3 (7%)	0.6 (2.9)	0.0 (0.2)
General practitioner	16 (24%)	13 (28%)	10 (15%)	6 (13%)	0.8 (1.8)	0.8 (1.3)
Midwife	1 (2%)	1 (2%)	7 (11%)	22 (48%)	0.9 (0.7)	1 (4.9)
Counseling	7 (11%)	1 (2%)	7 (11%)	45 (98%)	0.8 (3.3)	0.7 (4.3)
Activity services	3 (5%)	4 (9%)	43 (65%)	4 (9%)	1 (3.5)	0.91 (4.10)
Drug and alcohol services	2 (3%)	0 (0%)	9 (14%)	45 (98%)	0.3 (1.5)	0 (0)
**Child service use**						
Inpatient episodes	2 (3%)	3 (7%)	13 (28%)	3 (7%)	0.0 (0.2)	0.1 (0.4)
Outpatient	13 (28%)	5% (11%)	8 (12%)	5% (11%)	0.9 (3.0)	0.7 (0.3)
Early help/children’s social care	14 (21%)	4 (9%)	10 (15%)	8 (17%)	3.8 (11.0)	3.8 (11.0)
Community mental health services	11 (17%)	8 (17%)	10 (15%)	6 (13%)	1.5 (6.4)	2.6 (6.8)
Speech and language	3 (5%)	3 (7%)	15 (33%)	8 (17%)	0.6 (3.4)	0.6 (3.4)
General practitioner	5 (8%)	8 (17%)	14 (21%)	9 (20%)	0.2 (0.6)	0.2 (0.6)
Counseling	4 (6%)	1 (2%)	11 (17%)	11 (24%)	1.2 (6.6)	1.2 (6.6)

### Stop-go criteria

Within the feasibility trial, we met the success criteria for: the number of local authorities recruited; percentage of eligible parents consenting to the trial; and percentage of participants receiving the brief alcohol intervention (shaded green—Table 4). The extended brief intervention did not meet the success criteria (red—Table 4). Follow-up rates were marginal (amber—Table 4). The success criteria relating to both participant consent rates and follow-up varied by recruitment phase. While the 12-months follow-up success criteria were met within Phase 3, the participant consent rate within this phase became marginal. However, it should be noted that the a-priori success criteria were based upon numbers of parents refusing to give consent during an in-person interaction with their allocated social care worker. In Phase 3, we have taken non-response to a postal invitation to consent to the trial to be a refusal to give consent, which may relate more to the passive method that acceptability to parents of participating in a brief alcohol intervention trial.

**Table 4:** Success criteria.

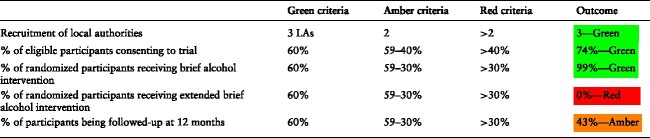

### Qualitative process evaluation

Interviews were conducted with *n* = 10 parents (*n* = 4 males; *n* = 6 females) and *n* = 10 practitioners (*n* = 3 males; *n* = 7 females) who participated in the trial. Parent’s AUDIT-C score ranged from 6 to 12. Practitioners in the sample had recruited between 0 and 7 participants. The mean duration of the parent interviews was 49 min (range: 17–100 min) and practitioner interview mean 31 min (range 15–52 min).

Trial processes were generally considered to be acceptable to both parents and practitioners. Most practitioners reported that they considered it appropriate for an early help and/or children’s social care practitioner to ask a parent about their alcohol use. Practitioners who had not recruited parents to the trial highlighted that they had screened parents on their caseload however these parents reported consuming alcohol below risky levels. Practitioners mostly commented that they considered this to be an accurate reflection of the parent’s consumption however occasionally practitioners questioned honesty of disclosure. There was a recognition that the context of safeguarding may introduce a level of fear for some parents, and that the timing of questions about a parent’s alcohol use within the overall professional relationships was important.

*“I think there are a lot of families out there… I think they're fearful of us initially…but I think once you get in and they see what you can offer them, most families do want that. It’s getting to that point” (children’s social care practitioner).*

All parents reported that they were accepting of the practitioner asking about their alcohol consumption. Indeed, some parents said that they had welcomed the opportunity to discuss their drinking. Where this was the case, parents reported that this provided an opportunity to reflect on their consumption levels in a way that was helpful to them.

*“[Early help practitioner] and I did have a really good bond, a really good relationship…it was like, actually if she is mentioning it then maybe I need to sort it out” (mother, early help).*

Practitioners randomized to provide brief advice reported feeling varying levels of competence at delivering the brief intervention. While the training they had received was generally considered to be sufficient, practitioners reported that they encountered too few parents who were risky alcohol users to enable these conversations to become routine. They highlighted that they had lacked the opportunity to provide brief advice in the period directly after the training and, therefore, became hesitant about delivering the intervention.

*‘If we were using it more and more frequently, I think it would be easier for people to embed. It's like everything. Things scare me when I first pick up a referral form and I think, "Oh." It's like, "I don't know what I'm filling in. Am I delivering this in the right way?" (Child protection social worker).*

Parents and practitioners were largely positive about the brief advice intervention however both groups reflected that the information about risks to children were often considered by parents to be ‘not relevant’ to the levels of alcohol consumption many of the parents reported. A focus upon more immediate, day-to-day impacts, were suggested as a preferred focus within the brief advice intervention.

*“Maybe if it could be worded differently from ‘risks for child’, or maybe thinking about how people would feel, they might think, oh if I say there is a risk, then they might have the kid taken off us. But thinking instead about stress in the mornings, my kids probably were late because I was running late because I’d had a drink the night before” (mother, early help).*

Similarly, parents and practitioners reported that the reference to a “safety plan” within the intervention, wherein parents either planned to reduce their alcohol use or implement practices which reduced risk to themselves or their children, was received negatively by parents. Parents experienced this to be judgemental and were resistant to the suggestion that their current alcohol practices were “unsafe”.

*“I didn’t think safety is like, well because the children weren’t at risk. Something along the lines of ‘plans for the future’ would be better” (mother, early help).*

## Discussion

This pilot feasibility provides important learning for trials of brief alcohol intervention in this novel setting. While the trial successfully recruited eligible parents in contact with early help and children’s social care services, this task was not without challenge. Two substantial changes to the recruitment strategy were required to achieve the sample. Parents were largely accepting of alcohol screening and brief intervention, however most practitioners found it difficult to integrate trial procedures within their routine practice. While some practitioners approached all parents on their caseload and recruited large numbers, others reported obstacles including workload pressure, sensitivity of the topic and a lack of confidence in intervening. When approached however, most eligible parents provided consent to participate in the trial. This suggests that for a definitive trial to be feasible, consideration must be given to how parents are first approached and recruited into the trial. Postal recruitment was both feasible and able to reach large numbers of parents who may otherwise not be identified. Within our trial, Phase 3 relied upon postal recruitment and therefore experienced lower consent rates, which is typical of such methods. However, twice as many fathers were recruited via postal invitation than other methods. Research has shown that family services often focus upon mothers as the primary caregiver. Fathers are typically overlooked due to viewing them as less important to child outcomes (Daniel and Taylor 2001), despite both father’s and mother’s alcohol use being found to be associated with adverse child mental health and substance use outcomes (McGovern *et al*. 2023).

Our trial found that participating parents were accepting of brief alcohol advice; however, it was not feasible to offer parents an appointment for extended brief interventions within local specialist alcohol services. This provides key learning for future trial design, which should examine only opportunistically delivered brief interventions. Previous research has demonstrated that parents who are risky alcohol users do not consider alcohol treatment services to be appropriate to their needs (Forrester and Harwin 2006; Alderson *et al*. 2022). It was notable however that within Phase 1 of recruitment, practitioners randomized to the extended intervention recruited the most parents into the trial. This finding reinforces our recent research that practitioners within the child safeguarding pathway prefer more intensive intervention (McGovern *et al*. 2022b). Further, the practitioners within our linked qualitative study described how parents may need time to develop a trusting relationship with a practitioner before being able to discuss their alcohol use freely. It is therefore likely that for brief alcohol interventions to be acceptable and feasible, a more intensive and flexible structure provided to parents outside of specialist alcohol provision may be required.

Retention of parents at follow-up varied by the approach taken with up to 63% of parents being followed-up at 12-months. This is below optimum levels, with the potential to introduce bias within a future trial to estimate effectiveness. Loss to follow-up occurred when follow-up was reliant upon making telephone contact with parents. While more active approaches to follow-up are generally found to be more successful in trials, it may be that parents in contact with early help and children’s social care services prefer the opportunity to complete questionnaires anonymously, and at a time that is convenient within the family routine. Postal follow-up may therefore enhance retention. While effort was made to minimize participant burden, questionnaire completion often exceeded 30–45 min. Health economic data collection highlighted the comparatively low service usage of this population as well as high proportion of missing data with 98% missing data to some questions. Simplification of the client service usage questionnaire may offer further opportunity to reduce participant burden and improve retention. Increasing the value of the “thank you voucher” to better compensate for the time taken to complete the questionnaire may also provide a proportionate incentive to enhance retention at follow-up.

## Limitations

This is the first feasibility RCT of alcohol brief interventions with parents in contact with social care services, and therefore, this represents an important advancement in the field. However, the trial has limitations. The trial was conducted in the North-East of England and may require some modification if tested in other geographical areas. Only parents aged 18 years and above were eligible for participation and therefore findings may not generalize to younger parents. The trial was on-going during the COVID-19 pandemic and therefore experienced disruption during periods of national lockdown which may have contributed to recruitment and retention difficulties. Within the qualitative process evaluation, we did not interview any parents that were randomized to the extended brief intervention, limiting our understanding of why this intervention was not accessed by parents.

## Conclusion

The findings of the pilot feasibility trial suggest that it may be feasible to conduct a randomized controlled trial of brief alcohol interventions to parents in contact with early help and social care, providing barriers to recruitment can be adequately addressed. The TLFB30 was found to be a suitable tool to measure the primary outcome of heavy episodic drinking. Measures to reduce expected attrition rates should be investigated further.

## Data Availability

Data from the feasibility trial is avilable from the lead author on request.
